# *mcr-1* Colistin Resistance in ESBL-Producing *Klebsiella pneumoniae*, France

**DOI:** 10.3201/eid2305.161942

**Published:** 2017-05

**Authors:** Yvan Caspar, Mylène Maillet, Patricia Pavese, Gilles Francony, Jean-Paul Brion, Marie-Reine Mallaret, Richard Bonnet, Frédéric Robin, Racha Beyrouthy, Max Maurin

**Affiliations:** Centre Hospitalier Universitaire Grenoble-Alpes, Grenoble, France (Y. Caspar, M. Maillet, P. Pavese, G. Francony, J.-P. Brion, M.-R. Mallaret, M. Maurin);; University Grenoble-Alpes, CNRS, Grenoble; Centre Hospitalier Universitaire Clermont-Ferrand, Clermont-Ferrand, France (R. Bonnet, F. Robin, R. Beyrouthy);; Centre National de Référence de la Résistance aux Antibiotiques, Clermont-Ferrand (R. Bonnet, F. Robin, R. Beyrouthy);; Université Clermont Auvergne, Clermont-Ferrand (R. Bonnet, F. Robin, R. Beyrouthy);; Institut National de la Santé et de la Recherche Médicale, Clermont-Ferrand (R. Bonnet, F. Robin, R. Beyrouthy);; Institut National de la Recherche Agronomique, Clermont-Ferrand (R. Bonnet, F. Robin, R. Beyrouthy)

**Keywords:** mcr-1, Klebsiella pneumoniae, bacteria, mcr-1 coliitin resistance, colistin, polymyxin, antimicrobial resistance, multidrug resistance, extended-spectrum β-lactamase, ESBL, chronic granulomatous disease, fungal meningitis, meningitis/encephalitis, infection control, France

## Abstract

We report intestinal carriage of an extended-spectrum β-lactamase−producing *Klebsiella pneumoniae* strain with high-level resistance to colistin (MIC 24 mg/L) in a patient in France who had been hospitalized for fungal meningitis. The strain had the *mcr-1* plasmid gene and an inactivated *mgrB* gene, which are associated with colistin resistance.

Resistance to colistin in gram-negative bacteria stems mainly from structural modifications of bacterial lipopolysaccharide. These modifications include addition of 4-amino-4-deoxy-l-arabinose or phosphoethanolamine caused by chromosomal mutations in genes encoding the 2-component systems PhoPQ and PmrAB, or mutations in the *mgrB* gene, a negative regulator of PhoPQ (*1*).

The recent discovery of a horizontally transferable plasmid-mediated *mcr-1* gene encoding a phosphoethanolamine transferase is a cause for concern, but few *mcr-1*−positive clinical strains of *Klebsiella pneumoniae* have been reported so far in Europe (*2*). Colocalization of carbapenemases or extended-spectrum β-lactamase (ESBL) genes and the *mcr-1* gene on the same plasmids is of concern because it might lead to pandrug resistance (*1*,*3*). We report *mcr-1* colistin resistance in ESBL-producing *K. pneumoniae* isolated from a patient in France.

The patient was a 38-year-old man who had chronic granulomatous disease that was diagnosed when he was 8 months old. Since then, he has had several minor and major diseases and conditions, including primitive femoral osteitis, hepatic abscesses, disseminated candidiasis, and bacteremia, which required several treatments with antimicrobial drugs. However, the patient was never given colistin.

In April 2016, he was hospitalized for surgical removal of a thyroid abscess. Fungal cultures of the abscess grew *Aspergillus fumigatus*. Despite antifungal treatment with amphotericin B and flucytosine, fungal meningitis, cerebral arterial vasospasm at the Willis polygon, and hydrocephalus developed. The patient also received immunosuppressive therapy (methylprednisolone and anakinra) and empiric antimicrobial drug therapy, including cotrimoxazole, clindamycin, meropenem, and vancomycin successively.

In August 2016, systematic culture of a rectal swab specimen showed an ESBL-producing strain of *K. pneumoniae*; 2 previous rectal screenings showed negative results. The strain was resistant to colistin (MIC >4 mg/L). Resistance was determined by using a broth microdilution assay (BD Phoenix Instrument; Becton Dickinson, Franklin Lakes, NJ, USA) (Technical Appendix). An MIC of 24 mg/L was found for polymyxin B by using the E-test method (Polymyxin B E-test strip; bioMérieux, Marcy L’Etoile, France).

The strain was sent to the French National Reference Center for Antibiotic Resistance in *Enterobacteriaceae* (Hôpital Gabriel Montpied, Clermont-Ferrand, France), which confirmed phenotypic resistance to colistin and identified the *mcr-1* gene by using PCR and previously described primers (*2*). Whole-genome sequencing showed that the *K. pneumoniae* strain had genotype ST15 and confirmed the presence of the *mcr-1* gene on a 33,303-kb transferable plasmid of incompatibility group IncX4 (Technical Appendix). This plasmid differed by only 4 mutations from *mcr-1.2*−encoding plasmid pMCR-1.2.IT (GenBank accession no. KX236309) previously characterized in Italy (*4*). Conjugation of the plasmid into *Escherichia coli* K12 conferred colistin resistance (MIC increased from 0.25 mg/L to 4 mg/L) to the *E. coli* strain.

Other resistance genes were also identified (Table), including the ESBL-encoding gene *bla*_SHV106_ (Technical Appendix). None of them were localized with the *mcr-1* gene on the IncX4 plasmid. Moreover, insertion of mobile element *IS5* in the *mgrB* gene was detected, which is also associated with colistin resistance (*5*). No mutations were found in the *prmA*, *prmB*, *phoP*, and *phoQ* genes.

**Table:** Resistance genes identified by whole-genome sequencing of an ESBL-producing *mcr-1*−positive *Klebsiella pneumoniae* strain isolated from a 38-year-old man, France*

Resistance gene	Target antimicrobial drug
*mcr-1* and inactivation of *mgrB* by *IS5* insertion	Colistin
*bla*_SHV-106_	β-lactams
*aac*(3)-IId and *aad*A16-like	Aminoglycoside
*aac*(6′)Ib-cr	Quinolone and aminoglycoside
*fos*A5	Fosfomycin
*sulI* and *folP*	Sulfonamide
*dfr*A27	Trimethoprim
*tetD*	Tetracycline

*ESBL, extended-spectrum β-lactamase.

There is currently no commercial medium to screen gram-negative bacteria harboring the *mcr-1* gene. Nordmann et al. (*6*) described an in-house SuperPolymyxin medium composed of eosin methylene blue agar, 3.5 mg/L of colistin sulfate, 10 mg/L of daptomycin, and 5 mg/L amphotericin B, which showed excellent sensitivity and specificity. Colistin resistance can be confirmed within 2 h by using an in-house rapid polymyxin Nordmann-Poirel test (*7*). The *mcr-1* gene can be rapidly detected by real-time PCR of DNA extracts obtained from bacterial strains or directly from stool samples (*2*,*8*,*9*).

We obtained subcultures of the strain from the patient on Columbia CNA agar containing 10 mg/L of colistin and 15 mg/L of nalidixic acid and 5% sheep blood (CNA^+^; bioMérieux) but not on Thayer-Martin agar medium containing unknown concentrations of vancomycin, colistin, amphotericin B, and trimethoprim (VCA3; bioMérieux). Lack of growth on this medium might be related to a high colistin concentration or the presence of vancomycin, which can potentiate colistin activity (*6*). Further investigations using CNA^+^ medium did not identify intestinal carriage of ESBL-negative but *mcr-1*−positive enterobacteria in the index case-patient. On the basis of these results, rectal screening of 39 contacts was performed by using an ESBL-screening medium (BLSE agar [MacConkey agar and Drigalski agar]; bioMérieux). All of the tests showed negative results.

The origin of the *mcr-1* strain remains unknown. Nosocomial acquisition cannot be ruled out because colistin-resistant strains harboring the *mcr-*1 gene might have been isolated in the hospital but not identified because this resistance mechanism was initially reported in February 2016. Food might also be incriminated (*1*); one study identified a 21% *mcr-1* prevalence among ESBL-producing *E. coli* in calves in France (*10*).

Multiple antimicrobial drug therapy for this patient might have selected for this multidrug-resistant bacteria. The presence of a plasmid containing the *mcr-1* and ESBL or other resistance genes in the same strain might be involved in selection of colistin-resistant strains during administration of any ineffective antimicrobial drug (*3*). Development of efficient tools for rapid detection of *mcr-1*−harboring strains should be a priority to prevent dissemination of these strains in hospital settings.

Technical Appendix Additional information on *mcr-1* colistin resistance in extended-spectrum β-lactamase–producing *Klebsiella pneumoniae*, France.
