# Supplementary material for: mcr-1 Colistin Resistance in ESBL-Producing Klebsiella pneumoniae, France
Source: Emerg Infect Dis. 2017 May;23(5):874–6. doi: 10.3201/eid2305.161942 (PMC5403025; doi:10.3201/eid2305.161942)
Supplement: Technical Appendix — Additional information on mcr-1 colistin resistance in extended-spectrum β-lactamase–producing Klebsiella pneumoniae, France. [file 16-1942-Techapp-s1.pdf]

Article DOI: http://dx.doi.org/10.3201/eid2305.161942

mcr-1 Colistin Resistance in ESBL-Producing *Klebsiella pneumoniae*, France

Technical Appendix

Sequencing of the *Klebsiella pneumoniae* Strain Genome and Plasmid Carrying the *mcr-1* Gene

We used a whole-genome sequencing method (Illumina, San Diego, CA, USA) with 50-bp paired and 60× coverage. Gaps in the plasmid carrying the *mcr-1* gene were filled by using PCR and Sanger sequencing.

Characteristics of the SHV-106 Plasmid

Whole-genome sequencing identified a 57-kb plasmid that belonged to incompatibility group IncR and carried the $bla_{SHV-106}$ gene. Genomic data were confirmed by extraction of plasmids according to the method of Kado and Liu (*1*) and hybridization with SHV and IncR probes.

Antimicrobial Drug Susceptibilities of the *K. pneumoniae* Strain

Antimicrobial drug susceptibilities were determined by using the BD Phoenix Instrument (Becton Dickinson, Franklin Lakes, NJ, USA). The strain showed susceptibility to amoxicillin/clavulanate (MIC 8/2 mg/L), piperacillin/tazobactam (≤4/4 mg/L), temocillin (8 mg/L), cefoxitin (≤4 mg/L), cefepime (≤1 mg/L), aztreonam (≤1 mg/L), ertapenem (≤0.25 mg/L), imipenem (≤0.25 mg/L), meropenem (≤0.125 mg/L), amikacin (≤4 mg/L), tigecycline (1 mg/L), and fosfomycin (32 mg/L); intermediate susceptibility to ticarcillin/clavulanate (16/2 mg/L) and ceftazidime (2 mg/L); and resistance to ampicillin (>8 mg/L), piperacillin (>64 mg/L), ceftriaxone (4 mg/L), tobramycin (>4 mg/L), gentamicin (>4 mg/L), nalidixic acid (>16 mg/L), ciprofloxacin (>1 mg/L), levofloxacin (>2 mg/L), norfloxacin (> 2 mg/L), colistin (>4 mg/L), and trimethoprim/sulfamethoxazole (>4/76 mg/L).

Reference

1. Kado CI, Liu ST. Rapid procedure for detection and isolation of large and small plasmids. J Bacteriol. 1981;145:1365–73. PubMed
